# Unraveling the skin; a comprehensive review of atopic dermatitis, current understanding, and approaches

**DOI:** 10.3389/fimmu.2024.1361005

**Published:** 2024-03-04

**Authors:** Moeina Afshari, Martina Kolackova, Michaela Rosecka, Jarmila Čelakovská, Jan Krejsek

**Affiliations:** ^1^ Department of Clinical Immunology and Allergy, Faculty Hospital and Medical Faculty of Charles University, Hradec Králové, Czechia; ^2^ Department of Dermatology and Venereology, Faculty Hospital and Medical Faculty of Charles University, Hradec Králové, Czechia

**Keywords:** atopic dermatitis, nano-dermatology, skin immunology, epidermal barrier dysfunction, immune dysregulation, nano-delivery systems, filaggrin gene mutations, Th Lymphocytes

## Abstract

Atopic dermatitis, also known as atopic eczema, is a chronic inflammatory skin disease characterized by red pruritic skin lesions, xerosis, ichthyosis, and skin pain. Among the social impacts of atopic dermatitis are difficulties and detachment in relationships and social stigmatization. Additionally, atopic dermatitis is known to cause sleep disturbance, anxiety, hyperactivity, and depression. Although the pathological process behind atopic dermatitis is not fully known, it appears to be a combination of epidermal barrier dysfunction and immune dysregulation. Skin is the largest organ of the human body which acts as a mechanical barrier to toxins and UV light and a natural barrier against water loss. Both functions face significant challenges due to atopic dermatitis. The list of factors that can potentially trigger or contribute to atopic dermatitis is extensive, ranging from genetic factors, family history, dietary choices, immune triggers, and environmental factors. Consequently, prevention, early clinical diagnosis, and effective treatment may be the only resolutions to combat this burdensome disease. Ensuring safe and targeted drug delivery to the skin layers, without reaching the systemic circulation is a promising option raised by nano-delivery systems in dermatology. In this review, we explored the current understanding and approaches of atopic dermatitis and outlined a range of the most recent therapeutics and dosage forms brought by nanotechnology. This review was conducted using PubMed, Google Scholar, and ScienceDirect databases.

## Introduction

Atopic dermatitis (AD), also referred to as atopic eczema, is a phenotypically heterogeneous chronic inflammatory skin disease. It typically arises due to environmental triggers in individuals who are genetically predisposed to the disease. AD is characterized by pruritus, particularly worsening at night, dry and leathery indurated skin, covered by intensively itchy papules which may release clear fluid when scratched. Additionally, xerosis, the term used for roughness of skin, and ichthyosis, a category of skin keratinization disorder, are the two terms often used with AD (1). The prevalence of AD is about 20% in childhood and 1-3% in adults, with female predominance, following a bimodal curve that peaks at early childhood and middle-aged adult population (2). AD was ranked 15th in the global burden of disease study in 1990-2017 among all nonfatal diseases, and ranked 1st among skin diseases measured in disability-adjusted life-years (DALYs) (3).The treatment of AD requires frequent physician visits and treatment compliance. The Out of pocket cost related to AD in the USA is $600 on average per person per year (4). AD is a member of a triad known as the “atopic triad”, along with allergic rhinitis and bronchial asthma. Recent studies indicate a sequential development of atopic disorders, referred to as atopic march, including asthma, allergic rhinitis, and food allergies in later childhood closely associated with infantile AD (5).

AD can be classified based on the age of onset. Pediatric patients are categorized into three main groups. The “very early onset” group includes patients who manifest their first signs of the disease as early as three months old. A significant portion of these patients experience complete remission by the age of two. This group accounts for more than two-thirds of all cases of AD. Patients aged between two and six years fall into the “early onset” category, while those patients between six and fourteen years are considered to have a “childhood onset” of AD. The “adolescent onset” group includes patients aged between fourteen and eighteen with limited available data. In contrast, adults are categorized into two main groups. The “adult-onset” group comprises patients between the ages of twenty and sixty, while the “very late onset” group includes patients over the age of sixty. The majority of adult patients are women with limited spectrum of sensitization (6).

In elder patients, it is recommended to carefully differentiate the potential diagnosis as other medical conditions, such as scabies, psoriasis, erythroderma, and cutaneous T-cell lymphoma, may manifest similarly to those of AD during their early stages. The diagnosis of AD relies on clinical observations as there are no validated, specific laboratory assays. In case of questionable or borderline diagnoses, skin biopsies may be recommended (7).

According to the diagnostic criteria established by the United Kingdom working party, diagnosis of AD requires the presence of itchy skin with three or more minor criteria. These minor criteria include flexural dermatitis or skin lesions, a history of generalized dry skin, asthma and the presence of a rash before the age of two (8). Noteworthy, these criteria are not applicable in very young children particularly infants. In infants and younger pediatric patients, the condition often affects extensor surfaces of extremities excluding diaper area, face, neck, and scalp. Papules on cheeks and cradle cap are commonly seen in infants in the first few months after birth (9). With further maturation into adolescence and adulthood, lesions tend to get fixated to more classical areas, namely the head, hands, neck, and flexural areas. Eyelid’s AD is also commonly seen in adult female patients. Clinicians acknowledge that, regardless of the specific phenotype of AD, pruritus remains the number-one complaint and first sign of AD in 87% of patients. The insomnia and anxiety stemming from excessive itch are the initial steps towards subsequent social and psychological burdens. The unpleasant sensation forces patients to scratch their skin, also raising the risk of skin barrier disruption, the likelihood of superimposed infections, and the sensation of pain (10). In cases of AD, hyperactivity of the nerves surrounding the atopic loci has been documented which may account for tingling and burning sensation. The density and diameter of nerves distribution was much higher in these patients compared to healthy controls (11).

Besides clinical features which aid us in accurate diagnosis of AD, validated scoring systems such as EASI (Eczema Area and Severity Index) and SCORAD (SCORing Atopic Dermatitis) subjectively and objectively stratify the severity of the disease into mild, moderate, or the severe forms (12). For many years, it was believed that clinical manifestations of AD were consistent across all ethnicities. However, recent studies suggest that the variation in transcriptomic profile of Asian patients, particularly in Th17 lymphocytes polarization, accounts for the difference in the clinical picture of chronic inflammation in Asians compared to Caucasians (13). Another notable difference is the mutation of filaggrin gene in Africans and Caucasians, as filaggrin deficiency is not observed in most Africans but is prevalent among Caucasian patients with AD (14).

The concept of precision medicine coupled with society’s growing demand to prevent or treat diseases in the most cost-effective manner, has led researchers to further investigate individual’s sensitization profile or detect biomarkers which predict the severity and identify patients before any clinical signs appear. One practical method to assess individual’s sensitization profile in AD is to assess total IgE and particularly allergen-specific IgE against common allergens such as food allergens or pollen allergens, and calculate the ratio of specific to total IgE (15). Measuring serum or plasma biomarkers is also promising approach proposed for diagnosis of AD.

## Skin; immunological perspective

The skin, the largest organ in the human body, originates embryologically from the surface ectoderm, neural crest, and mesoderm. Skin serves as an insulator of the internal surface from the external world by shielding against invasion of pathogens and toxins. The thickness of skin varies across different body sites and protects against physical trauma and chemical injuries. Additionally, skin preserves water and electrolytes and contributes to the body’s temperature regulation. In conjugation with the endocrine system, skin plays a role in hormone production (16). Various types of cells, including some originating from bone marrow, are distributed throughout the skin, and serve as immune or non-immune cells. The skin compromises three main layers, in order from top to base, the epidermis, the dermis, and the hypodermis. The epidermis itself is further divided into stratum corneum, lucidum, granulosum, spinosum and basale.

Keratinocytes, which represent the most abundant cell type in the skin and the fundamental building blocks of the epidermis, continually undergo proliferation and migration to maintain a balance between the ongoing terminal differentiation and desquamation occurring at the uppermost layer of epidermis. The transformation of keratinocytes into corneocytes are known as cornification. Corneocytes are a-nucleated keratinocytes with high keratin content and no inner organelles, covered in the cornified envelope, ceramides, free fatty acids, and cholesterol to form the first line of defense. Changes in the quantity and composition of ceramides are linked to pathogenesis of AD (17). Keratinocytes together with neutrophils and natural killer cells(NKs), are major producers of antimicrobial peptides (AMPs). AMPs protect skin against infections and inflammation, by forming a chemically stable cover on the skin surface. Patients with AD have been documented to exhibit reduced levels of AMPs, specifically cathelicidin and β-defensins, exposing them to greater risk of Staphylococcus aureus infections (18). Keratinocytes express Toll-like receptors (TLRs), which activate Th1 lymphocytes to produce interferon-gamma (INF-γ) and pro-inflammatory interleukins (ILs) such as IL-1 β and IL-18, which further facilitate leukocyte migration to the skin (19).

Langerhans cells (LCs) are resident immune cells of the epidermis. They are resident macrophages which function like dendritic cells. They operate as a binary system in response to foreign invaders. LCs are primarily known for their negative regulatory immune actions and tolerization (20). Research has shown that LCs induce CD4+ regulatory T lymphocytes, and promote tolerance in CD8+ T lymphocytes, and cause production of IL-10 by Langerhans cells which suppress contact dermatitis (21). These processes represent rapid and nonspecific primary responses known as the innate immune response, thus LCs are classified as innate immune cells. Langerhans cells possess long processes called dendrites; these processes are elongated to reach the epidermal tight junctions allowing contact to pathogens via pattern recognition receptors (PRRs). After internalization of pathogens associated molecular patterns (PAMPs), they are processed into antigenic fragments subsequently bound to human leukocyte antigen II (HLA-II) molecules, this happens during migration of LC into skin draining lymph nodes through dermal lymphatic vessels. Finally, they will present the processed linear antigenic peptide bounds by major histocompatibility complex (MHC)class II molecules to the T lymphocytes, thereby initiating an adaptive immune response (22). Within the epidermis, several other cell types can be found, including pigment-producing melanocytes and sensory Merkel cells for skin sensation. Memory T lymphocytes may also be found in healthy epidermis, although much more extensive infiltration of T lymphocytes and neutrophils are seen in AD (23).

Dermis, the second and thickest layer of the skin, hosts various cells that contribute to the immune system. There are couple of non-immune cells in the dermis which play a dual role in maintaining dermal integrity and providing a pathway for transportation of immune cells. Fibroblasts in the dermis produce structural proteins that act as a supporting framework. Endothelial cells, lining the skin’s blood vessels, express adhesion molecules and produce cytokines facilitating oriented passage of immune cells to the upper epidermis.

Dermal dendritic cells (dDCs) are positioned just beneath the epidermal-dermal junction and possess fewer dendrites compared to Langerhans cells. This reduction result in greater mobility for immune interactions. Dermal dendritic cells express epithelial-cell adhesion molecules as well as IL-10. Their distinctive function involves stimulating B cells to produce various classes of immunoglobulin, such as IgM, for immunosurveillance against pathogens through cytokines and chemokines productions. This activity highlights their significance in both innate and adaptive immunity of the dermis (24, 25).

Monocytes are found circulating in the bloodstream serving as a precursor for macrophages. Monocytes remain in a state of readiness waiting for a cue to be recruited into the dermis. Upon differentiation to macrophages, they transform to highly mobile phagocytic cells capable of initiating immune response by PRRs and release of cytokines (26). Recent studies have provided evidence that in the normal state, monocytes function as tissue antigen presenters to T lymphocytes. Additionally, skin-resident macrophages are established in the dermis during embryonic development and are renewed only by *in situ* proliferation (27).

Another large group of innate immune cells occupying the dermis are granulocytes. Neutrophils, basophils, eosinophils, and mast cells contain granules filled with proteases and antimicrobial peptides. The degranulation of basophils and mast cells is IgE-dependent. The crosslinking of FcϵRI receptors occupied by IgE immunoglobulins, which are recognizing specific allergens, causes the release of content of cytoplasmic granules. Overall, when macrophages and dendritic cells get activated, they recruit granulocytes to the site of encounter with invaders; neutrophils are the initial cells to get recruited (28).

Mast cells represent another cell type present in the dermis. These cells contain histamine and act as effector cells in IgE-mediated hypersensitivity reactions (29). Mast cells are tryptase and chymase positive. These enzymes are important in degrading a pathway that allows invasion of activated immune cells to the epidermis (30). In addition to enzymes production, mast cells activate other immune cells by direct contact or cytokine production. In AD, mast cells, along with other immune cells such as NKs, eosinophils, and basophils, produce IL-4, which results in overt Th2 polarization of the antigen stimulated naïve CD4 lymphocytes (31). In a study performed on normal human keratinocytes and skin equivalent models, the inhibitory effect of INF-γ produced by mast cells on tight junctions, specifically claudin-1 was revealed, resulting in compromised barrier integrity (32). Mast cells engage with other cells through expression of TLRs and HLA I and II. Their production of co-stimulatory molecules such as CD80 or CD86 further facilitates antigen presentation. The interaction of mast cells with Langerhans and dermal dendritic cells is pivotal for dendritic cells maturation, migration, and antigen presentation, accomplished through production of TNF-α and histamine. Mast cells are capable of inducing immune tolerance by production of IL-10, transforming growth factor-β (TGF-β) and an increase in the number of T-regulatory lymphocytes (33). TGF-β can modulate B lymphocytes activity by limiting the germinal center formation and promoting IgA class switching. Furthermore, TGF-β was found to be driving the fibrotic process following the damage caused by inflammation in AD (34).

The final category of innate immune cells in the dermis, known as NKs, share some characteristics that bridge both innate and adaptive immunity. They function as innate cells by detecting surface HLA-I molecules and operate on basis of PRRs. When the cellular expression of surface HLA-I is reduced, as seen in virus-infected or cancerous cells, the NKs become activated. Additionally, They can perform an antibody-dependent cytotoxic attack, by binding to the IgG-labelled pathogens or cancerous cells, releasing their intracellular granules or inducing apoptosis of these cells (35).

Dermis is the home to most adaptive immune cells in the skin. While adaptive immune cells travel through skin for surveillance, some such as memory T lymphocytes can also be found in healthy epidermis. T lymphocytes can be grouped to several subpopulations but the most extensively studied groups are cytotoxic T lymphocytes and T helper lymphocytes. Cytotoxic T lymphocytes, also known as CD8+ T lymphocytes, are the effector cells recognizing surface antigens represented by antigen presenting cells. Upon recognizing HLA I class molecules occupied by foreign antigenic linear peptide on the cell surfaces, a programmed cell death process is initiated (36). After effective antigen recognition, CD4+ T lymphocytes are clonally expanded and functionally polarized into different subsets, such as Th1, Th2, Th17 and Treg lymphocytes. Polarized T helper lymphocytes, support effector cells by producing cytokines and aiding in maturation of dendritic cells. The cytokines produced by T helper lymphocytes (CD4+) couple with another group of adaptive immune cells, known as the B lymphocytes. Recently there are new findings proving B lymphocytes are passively and actively participating in maintaining immunity in the skin through specific-antibodies production. Mature B lymphocytes, after antigenic stimulation, are clonally expanded and terminally differentiated into plasma cells. This process is tightly regulated by Th2 polarized T lymphocytes. Plasma cells which can produce specific antibodies, reside in lymph nodes, and bone marrow. Their secreted antibodies reach the target site by circulation. Recent data indicates that B lymphocytes are actively involved in both inflammation and immunosuppression within the skin. They produce proinflammatory cytokines and anti-inflammatory IL-10, highlighting the hemostatic role of B lymphocytes in host defense (37). B lymphocytes can also serve as very effective antigen presenting cells to T lymphocytes and important source of the spectrum of immunoregulatory cytokines. There are as many as 20 billion T and B lymphocytes in the skin, making the skin a potential candidate as a peripheral lymphoid organ (38).

## Immune dysregulation in atopic dermatitis

While the epidermal barrier impairment is commonly recognized as the initial trigger of AD, it is crucial to acknowledge the dysregulation of innate and adaptive immune system in the pathogenesis of AD. According to the “Inside-out” hypothesis, the barrier impairment in AD is the consequence of immune responses to irritants and allergens (39).

Innate immunity is expected to be the primary defense against the invaders of the skin layers, although it’s impairment is documented in the AD (40). It is also documented that patients suffering from AD are predisposed to infections as the process of pathogen recognition and invasion control is impaired (41).

Keratinocytes, Langerhans cells, stromal and endothelial cells, macrophages, and various other immune cells residing in the skin are equipped with PRRs which serve as initiator of epidermal immune reaction through recognition of pathogenic patterns known as PAMPs. PRRs are responsible for pathogen recognition by activating the innate immune system and antigen-specific adaptive immunity (42). PRRs encompass several families of receptors, such as TLRs. This support is unreplaceable for isotypic switching and somatic mutation of antigen stimulated B lymphocytes. Activation of downstream signaling pathways result in activation of various transcription factors, which are regulating several hundreds of genes with proinflammatory activities, promoting production of pro-inflammatory proteins. The inflammatory cytokines along with the antimicrobial peptides are the rapid primary responses against pathogens, however, in some instances these factors are unable to combat the pathogens sufficiently, alternatively, TLRs also shape the adaptive immune response by activating the maturation of dendritic cells as antigen presenting cells, and influencing T and B lymphocytes function to act as a second line of defense (43).

Inflammatory cytokines, such as IL-6 and TNF-α, can stimulate neighboring cells to produce chemokines and adhesion molecules to further recruit additional immune cells to the site of invasion. TLRs can also recognize both damage-associated molecular patterns (DAMPs), expressed by damaged skin cells, and pathogen-associated molecular patterns (PAMPs) expressed by invading pathogens. Such recognition enables innate and adaptive immune system activation as well as enhancing host defense molecules production such as AMPs and proinflammatory cytokines. Excessive TLRs activation can inadvertently cause T lymphocyte mediated autoimmunity, further predisposing individuals to undesired inflammation and skin conditions, such as AD. Keratinocytes, dermal fibroblasts, and Langerhans cells are equipped with PRRs and are also capable of production of AMPs and cytokines. When activated by DAMPs or PAMPs, these cells will recruit other immune cells including neutrophils, macrophages, and T lymphocytes to establish an adaptive immune response, and a memory for possible re-invasion (44).

Keratinocytes, the abundant cell type on the skin surface, are equipped with TLRs. TLR 2 and TLR 3 are the main receptors of pathogens in keratinocytes. As bacterial lipopeptides are recognized by TLR 2, proinflammatory cytokines such as IL-6 and tumor necrosis factor-alpha TNF-α are produced. In a study performed on Leiden epidermal models, it was proven that TNF-α alone or in combination with other cytokines, such as IL-4 or IL-13, decrease the length of long chain free fatty acid and ceramides leading to altered barrier function (45). TLR 3 on the other hand, ensures a normal barrier function by inducing expression and function of tight junction as well as ensuring re-epithelization, granulation, and vascularization for wound healing (19).

Dermal fibroblasts are equipped with TLR 2 and 4, allowing them to recognize bacterial lipopeptides and fungal pathogens, promoting proinflammatory cytokines production. They are also able to differentiate to preadipocytes upon dermal infection with Staphylococcus aureus. The role of adipocytes in prevention of further invasion of Staphylococcus aureus is still an ongoing area of research (46).

Dendritic cells of the skin and the LCs are dependent on TLRs for their functioning. TLRs enable these cells to present antigens to T lymphocytes and induce production of cytokines and chemokines. Upon detection of the PAMPs and DAMPs, they migrate to lymph nodes to prime T cells for further immune responses. Langerhans cells possess several TLRs, such as TLR 2,3,4,7 and 8. TLRs in LCs can produce both cytokines and chemokines such as proinflammatory cytokines, IL-12, IL-8, and CCL3. IL-12and IL-8 were further studied for correlation between skin samples levels or serum levels and the severity or progression of AD. IL-8 was elevated in stratum corneum samples of AD subjects with highest correlation to the severity scores (47). In another study, the measured serum sample level of IL-12 correlated with the severity score, SCORAD, in a concentration dependent manner (48). Chemokines such as CXCL9, CXCL10, and CXCL11, used as biomarkers of AD, are expressed when TLR3 is activated in the LCs (49).

AD has been associated with impaired TLR2 function, as evident by genetic polymorphism of TLR2 and the down-regulation of its expression in macrophages and peripheral blood mononuclear cells of the patients (50). In a research performed by Yu et al. on the mentioned cells, samples treated with TRL2 ligands produced less of Th1 and Th17 cytokines compared to Th2 cytokines, supporting the Th2 polarization in AD (51). Additionally, confocal microscopy of skin sections of AD patients revealed less TLR2 expressed in basal keratinocyte compared to the whole width expression in the normal skin (52). In normal skin, TLR2 activation in keratinocytes leads to an increase in the expression of tight junction proteins, such as claudin, and antimicrobial peptides such as cathelicidine. However decreased levels of tight junctions and AMPs are commonly observed in all individuals with dermatitis (53).

Thymic stromal lymphopoietin (TSLP), is a highly expressed cytokine in atopic dermatitis. Upon TSLP expression, the innate and adaptive immune system develops a connection to demonstrate an atopic reaction. TSLP is not confined to skin barrier surfaces, but it is also found in lungs and gastrointestinal tract (GIT). The TSLP receptors are also found on variety of cells, including dendritic cells and monocytes. TSLP exists in two isoforms, the one known as lfTSLP, has a long chain, while the latter is referred to as sfTSLP (54). TSLP-1 was found to be upregulated in lesioned skin of AD patients. Elevated TSLP-1 levels may be detected before development of any AD phenotypes, making TSLP-1 a candidate biomarker for early detection of AD. Studies involving both humans and mice have revealed the elevation of TSLP levels upon activation by TLRs ligands. TSLP is a key cytokine which triggers the Th2 cytokine associated inflammation in atopic diseases including asthma and allergic rhinitis (55). Keratinocytes and mast cells are among the main producers of TSLP in AD. Further, TSLP promotes the activation and migration of dendritic cells in the dermis. These dendritic cells produce Th2 attracting chemokines in order to notify the differentiation of naïve CD4 T lymphocytes to Th 2 lymphocytes (56). Furthermore, a study conducted on skin explants showed that TSLP induce allergic inflammation by inducing production of proinflammatory cytokines such as IL-4 and IL-13. In return, TSLP is further upregulated by TNF-α, IL- 1α, IL-4 and IL-13. The collaboration of these cytokines synergize and result in the initiating role of TSLP in allergic responses (57).

The severity of AD goes hand in hand with the higher cytokine expression. In a study performed on mice models, it was revealed that the absence of IL-4 enhances the epidermal barrier function and innate defense mechanisms (58). More recently, the association of IL-4 and IL-13 has been studied, owning to similarities in their course of action in AD. While both cytokines act on the IL-4Rα receptors, it was evident that IL-13 rather participate more peripherally compared to IL-4, which is expressed in lymph nodes by T lymphocytes. IL-13 is locally-expressed in the skin, down-regulating OVOL1-FLG pathway, which results in epidermal barrier dysfunction and increased trans-epidermal water loss (TEWL) (59). IL-13 and IL-4 reduce the production of AMP by keratinocytes, predisposing the skin to dysbiosis and penetration of pathogenic entities (60). Further, experiments on mice models have shown that IL-4 and IL-13 reduce the sensitivity threshold of sensory nerves to pruritogenic stimuli, such as alarmins, and can also directly induce itching behavior in the experimental models (61).

IL-5 modulates the clinical activity and severity of AD, through regulation of IgE synthesis and eosinophilic activation (62). IL-5, which is produced by Th2 lymphocytes, co-expressed with IL-4 and IL-13, can increase vascular permeability and muscular contractibility. IL-5 together with other chemo-attractants, such as CCL-11, draws eosinophils to vascular compartments, resulting in inflammation. IL-5 also prolongs eosinophils activities and survival by reducing their peripheral apoptosis and increasing their bone marrow genesis (63).

The studies on human skin and mice models have shown the over-expression of IL-31 and its receptor, IL-31RA, in dermatitis models. In mice models, IL-31 was identified as a pruritogen, independent of mast cells (64). Protease activated receptor 2 (PAR-2) activation cause IL-31 and TSLP release, which stimulate the immune cells, and the pruriceptive sensory nerve fibers to induce itch. In a study by Takaoka et.al, the overexpression of IL-4 and IL-31 were detected in the epidermis and hair follicles of the mice (65). Further, IL-31 stimulate sprouting and branching of sensory nerves, playing a major role in the chronic itch in AD, and the hyperkinesis and alloknesis phenomenon. Hyperkinesis and alloknesis both contribute to the itch-scratch cycle in AD, complicating the nature of pruritus (61). In another study on human skin affected by dermatitis, the addition of staphylococci toxin to the serum of the subject group produced higher IL-31 expression compared to the control group, further proving the role of staphylococci in overexpression of cytokines leading to dermatitis features (50).

IL-17 is a Th 17 lymphocyte cytokine, which was found to be increased in the peripheral blood of AD patients with high disease severity. IL-17 amplifies the production of IL-4 by Th2 lymphocytes, causing epidermal barrier dysfunction (66). It is a cytokine that is enhanced by superantigen staphylococcal enterotoxin B and negatively correlates with IgE levels and eosinophils count (67). The IL-23/IL-17 axis holds significance in chronic atopic dermatitis. Th17 cells, activated under the influence of IL-23 produced by immune cells and keratinocytes, produce IL-17A and F, IL-6 and TNF-α. IL-17 suppresses the synthesis of filaggrin in keratinocytes, impairing the skin integrity and barrier (68). Interestingly, in a study by Mizutani et.al, co-stimulation of keratinocytes and skin equivalent models with IL-17A resulted in restoration of tight junctions dysfunction induced by INF-γ (32).

## Pathogenesis of atopic dermatitis

Etiopathogenesis of AD is not yet very clear. Although genetics, environmental, and immunologic factors play a significant role in its development. In a recent meta-analysis, it was documented that the probability of children developing AD is increased by approximately 40% if they have at least one parent with atopic history. This probability rises even further to 60-80% when both parents suffer from atopic diseases (69). Furthermore, Genome-wide linkages studies have identified potential associations with AD on various chromosomes including chromosome 1,3,5,11,15, and17, despite no specific loci on genes were pinpointed (70).

Another aspect of genetics in AD are the filaggrin gene loss of function mutations. In short, filaggrin (FLG) is one of the key proteins in the terminal differentiation of keratinocytes and of the protective skin barrier (71). The skin uses by-products of FLG metabolism to produce natural moisturizing factors (NMFs), which are crucial for skin hydration and prevention of Staphylococcus aureus over-growth (72). FLG metabolism ends in the production of amino acids which may have a role in the acidification of skin pH and a factor facilitating the action of antimicrobial peptides. For many years, it was believed that deficiency of FLG is a single agent causing AD. Later it was proved that not all patients suffering from AD have FLG gene loss of function mutations, but most with barrier dysfunction are the carriers of the mutation. The deficiency of FLG was pronounced in Europeans and Asians who are carriers of two null FLG mutations. Approximately forty loss of function mutations in genes coding for FLG were found in affected populations, except in Africans, therefore the mutations in FLG gene were rather considered as predisposing factor for AD (73).

The word “filaggrin” is a short form for filament aggregation protein. FLG acts as an aggregator that compacts the keratin intermediate filaments, which are necessary in keratinization of skin cells. The differentiation process of keratinocytes to corneocytes is dependent on a calcium gradient. The calcium gradient is higher in the stratum granulosum compared to stratum corneum. Thus, the gradient difference drives the differentiation while the keratohyalin granules in stratum granulosum release their content, profilaggrin (74).

Profilaggrins, under the influence of serine proteases, become FLG monomers. Being the most important component of the cornified envelope, the FLG monomers cross link the intermediate keratin filaments to form bundles and promote dehydration of keratinocytes and subsequently cause the cellular flattening and collapse. The uppermost layer of epidermis is supported by a bilayer of lipid-rich extracellular matrix, resembling the analogy of mortar and bricks, where bricks are the corneocytes and the extracellular matrix is the mortar. The mentioned layer of cells are very efficient in protecting deeper layer of skin when they are intact (75).

Studies have shown that patients with FLG gene mutations, have marked falls in NMF concentration and keratohyalin granules. A generalized decrease in density of corneo-desmosomes along with disordered architecture of extracellular lipid matrix are also observed. The organization of keratin filaments, loading of lamellar body contents for secretion of lipids and enzymes, and an increase in pH, which favors inflammatory reactions, are also the consequence of FLG deficiency (76). FLG gene mutations are capable of recruiting immune cells to the site of invasion, as induced keratinocytes will produce more IL-1 (77). FLG deficiency was also linked to IL-33 up regulation in mice models (78). In another study, higher serum level of IL-33 was showed in AD patients compared to healthy controls (79). Further, in a study by Howell et.al, the effect of IL-4 and IL-13, which are Th2 inflammatory cytokines, on FLG expression was studied. The aim of the study was to determine if those cytokines modulate FLG expression during keratinocytes differentiation. The IFN-γ was used as Th1 cytokine control. Five days differentiation of keratinocyte in presence or absence of IL-4 and IL-13 resulted in significant reduction of FLG expression. In contrary, differentiation of keratinocytes, in the presence of IFN-γ, augmented the expression of FLG (80).

The increased barrier permeability, by FLG mutations, facilitates the passage of allergens to deeper layers of skin and subsequently increases the chance for the encounter of allergens with LCs, initiating a complex immune response. In a study performed by H. Kondo, the percutaneous sensitization through disturbed skin barrier was proved in the mice models, accompanied by predomination of Th2 mediated immune responses proving polarization in adaptive immunity (81). The local increase in the number of eosinophils and mast cells, as well as increased allergen specific IgE and total IgE, added to the increased TSLP-1 and IL-1α production by disturbed keratinocytes, further favors the immune polarization (82).

In a study carried out in Copenhagen on 411 children born to mothers with a history of asthma, a specific endotype of AD was documented. The FLG gene mutations predispose to an endotype with an earlier onset of the dermatitis with a more severe course (83). In another study carried out in Pennsylvania on African-American children, it was noted that mutation in FLG-2 was accompanied with a more persistent course of disease continuing to adulthood (84).

Familial or ethnic background affects the outcome of the disease, but the increased global prevalence of atopic diseases cannot be solely dependent on genetics. It is likely that the interplay between genetic predisposition and environmental exacerbation are the possible explanation for the increased prevalence, thus recognition of the environmental factors triggering the onset or recurrence of AD could be a window to prevention.

It was previously believed that prenatal exposure to cigarette smoke and maternal alcohol consumption may increase the likelihood of childhood atopies (85). Although more recent studies could not reproduce the same findings, passive exposure to cigarette smoke is proved to be associated with increased AD prevalence (86). Multiple studies on the effect of breastfeeding or hydrolyzed formula feeds and their duration with or without halting of solid foods has not shown a clear change in prevalence or outcome of AD (87). In contrary, several studies have proved the positive effect of maternal fish ingestion prenatally on lowering the chances of developing AD (88). Further, the postnatal ingestion of fish at one year of age has shown to decrease the prevalence of AD, hence increased dietary fish intake is advised (89).

The “hygiene hypothesis” is another theory regarding the effect of environment on the development of atopic diseases in children. In this theory it is proposed that children living in countries with lower socioeconomic status and rural living standards, who may be exposed to farm animals and unpasteurized milk, are less prone to development of AD. Exposure to dirt and pathogens such as helminths and Herpesviridae has shown to modulate the immune system, favoring less occurrence of allergic inflammations (90).

Although it was documented that exposure to bacterial endotoxins and early exposure to infections in daycare may be a protective factor against AD, not all contracted pathogens have positive protective roles. The respiratory syncytial virus (RSV) and herpes simplex virus (HSV) were documented to increase the risk of developing dermatitis (91). The hygiene theory is further supported by the study showing that prenatal exposure to antibiotics, increases the chances of developing AD, and exposure to probiotics and omega 3 fatty acids, decreases the probability. The early exposure to antibiotics harm the microbiota of skins and guts of fetuses and newborns. In contrast, the use of probiotics strengthen the microbiota (92).

Skin microbiota has always been a question to researchers pertaining to AD. The skin microbiota contribute to the fight against pathogens by inducing production of AMPs and maturating the innate immune system from day one of human life (93). The skin microbiota of newborns delivered vaginally is similar to the vaginal flora, further strengthening the microbial diversity of skin compared to newborns delivered by c-section. Therefore, infants may be predisposed to AD already at birth only according to the mode of delivery (94). Commensal species, such as Staphylococcus epidermidis, suppress Staphylococcus aureus over-growth and toxin production by inducing AMPs production by keratinocytes, through activation of Toll-like receptor 2 (TLR-2) and nuclear factor kappa B (NF-κB) signaling pathway. They are also able to abrogate NF-kB suppression induced by pathogenic species such as Staphylococcus aureus (95). The global influence of AD on lesioned and non-lesioned skin microbiota was studied by Clausen et al. proving that FLG mutation also contributes to the disturbance of the symbiotic balance (96).

Upon epidermal barrier dysfunction, the balance between commensal and pathogenic species is disturbed. The organic diversity is challenged as species such as Staphylococcus aureus colonize the skin, outnumbering the other commensal species. The disease progression in AD is tightly bound to pathogenic colonization. The severity and chronicity are among the consequences of pathogenic colonization (97). The pathogenic proteins of Staphylococcus aureus induce production of proinflammatory cytokines such as IL-4, IL-5, IL-6, IL-17 and TSLP in human and animal models (98). Staphylococcal protein A (SpA) can induce production of alarmin IL-33 by keratinocytes (99), which in turn reduces the expression of filaggrin and claudin-1 responsible for the skin barrier integrity and chronic itch (100). Th2 polarization and allergen sensitization predispose AD patients to a viral complication. AD patients display low INF-γ and its receptor, resulting in defective response to viral multiplication, as the T cell polarization is shifted from Th1 to Th2 (101). The impact of AD-related cytokines and Staphylococcus aureus on adult human keratinocytes was investigated. The study showed that the heat-killed Staphylococcus aureus and Staphylococcus epidermidis, in presence or absence of T Lymphocytes-derived cytokines, induce a distinctly different gene expression in keratinocytes. The cooperation of cytokines and Staphylococcus aureus contributed to the exacerbation of AD-associated transcriptomes, which provide us a new understanding about the role of Staphylococcus aureus in the AD flares (102).

Fungal sensitization is often observed among AD patients. Among fungal species are Candida albicans and Malassezia furfur. The sensitization may elicit IgE response along with cytokine production and self-reactive T lymphocytes stimulation (103). The molecular mimicry and cross-reactivity are thought to be the mechanism behind fungal aggregation resulting in atopic dermatitis (104).

Skin pH is another pivotal physical factor in the integrity of the skin. The skin pH is 4.7 on average (105). The acidic pH is the result of different intrinsic processes maintaining not only the acidity but also moisture and barrier integrity. It is proved that acidic milieu, is a protective factor for atopic dermatitis as it supports antimicrobial activity and barrier function (106). There are number of non-preventable factors which fluctuate the skin pH. Skin pH is age dependent; newborns are born with skin of neutral pH although by the age of one, the pH is decreased to more acidic pH (107). The elder’s skin pH is more basic, as there is ceramide deficiency and subsequent stratum corneum dehydration. This phenomenon may be the explanation to sudden increase in prevalence of AD in older age. Among the preventable causes of pH disturbances are the use of personal hygiene care products. All these agents act as an emulsifier of the skin surface lipid. Sodium lauryl sulfate and surfactants, constituents of many hygiene products, are known for their negative effect on skin surface as they increase TEWL (108).

Stratum corneum can be externally damaged by meteorological variables, such as low ambient humidity and extreme ambient temperatures. In AD, decreased ambient humidity exponentially increases the gradient of TEWL, leading to dryer skin (109). Although higher humidity hinders excessive TEWL, the increased perspiration provoked by humidity may worsen the severity in the established disease (110). There is a need for vaster epidemiological studies to explore the exact role of humidity in provoking or preventing AD. In a study in the Swiss high-mountain area of Davos, it was noted that increased temperature from very low to moderate temperature of +18°C, decreased the pruritus and severity of eczema (111). On the contrary, in an American study, it was noted that higher temperatures are associated with poorly controlled AD (112). Although individuals living in warmer countries are better exposed to eczema-protective factor, UV-light, the overall harm caused by excessive perspiration and subsequent Th2 activation may outweigh the benefits. The prevalence of AD could also be increased with the rise of absolute latitude. The observation in Australia showed that AD prevalence is higher among children living in the central and southern regions of Australia as these areas are further from the equator (113).

Human life is industrializing at an undoubtedly faster pace than expected. Air pollution is one of the many detrimental effects of industrialization and urbanization on human lives. Air pollution may also exacerbate skin barrier defects and heighten immune responses (114). In a study carried out on AD patients and healthy controls, the effect of nitrogen dioxide, which is shed from diesel engines in high concentration in urban areas due to heavy traffic, and formaldehyde, present in most indoor environments, was investigated to differentiate whether air pollution can further challenge skin barrier function. The study showed that upon exposure to nitrogen dioxide, the TEWL is increased in the eczematous skin at the state of barrier dysfunction compared to TEWL in eczema patients prior to the test and healthy controls (115). Other studies showed an increase in NF-κB production of mice models (116) and an increased production of IL-6 and IL-1β by cultured human keratinocytes, resulting in further induction of proinflammatory cytokines production (117, 118).

Recently researchers have focused on the interplay between food allergies or sensitization and AD. Although it is believed that AD preceded food allergies but not vice versa, the mechanism is not very clear. Number of studies have proved that patients with more severe phenotypes of AD, show more frequent diagnosis of food allergies or sensitization (119). It is believed that food sensitization could occur percutaneously. The impaired skin barrier in AD patients is a great entry point for food allergens via skin. As food sensitivity and allergy are two different entities with notable differences in severity; It may be recommended that children diagnosed with AD undergo some skin prick test or oral challenge test for common food allergens, to eliminated foods which may be triggering co-expression of food allergy and AD. These tests may prevent excessive food avoidance, which may cause nutritional issues in children (120).

Understanding the role of epidermal barrier in the pathogenesis of AD is crucial. The “Inside Out or Outside In theories” are still at the center of current research. It is not completely proven if barrier disruption causes the AD or the vice versa, nonetheless, the barrier dysfunction causes excessive TEWL and increased permeability to different irritants and pollutants to the deeper layers of the skin (121).

Diverse functions of epidermal barrier, from preventing excessive TEWL and microbial invasion to its photoprotective role is mostly mastered in the uppermost layer of the epidermis. There are exogenous and endogenous factors which alter the integrity, inevitably causing a disturbance in barrier integrity.

Skin desquamation is delicately controlled by different types of proteolytic enzymes, named as epidermal proteases, and their inhibitors. These enzymes hydrolyze peptide bonds of corneo-desmosomal adhesions and subsequently result in detachment of upper cellular layers of epidermis. Four families of proteases have been identified of which serine proteases are the most researched proteases found in the epidermis. Kallikrein-related peptidases (KLKs), a member of serine proteases family, account for most of the proteolytic function in the desquamation process. The up regulation of proteases can stimulate overexpression of proinflammatory cytokines through protease activated receptor 2 (PAR2), resulting in production of chemokines and cytokines involved in pathogenesis of AD (122). PAR2 receptors are also found on keratinocytes and sensory nerves. They play a major role in the production of neuropeptides, such as substance P and calcitonin gene-related peptides, which stimulate mast cells to release tryptase and induce non-histaminergic pruritus in AD (61).

The most important members of KLKs in the pathogenesis of AD are KLK-5,7 and 14. They are synthesized among their inhibitors, Lympho-epithelial Kazal-type-related inhibitor (LEKTI), in the stratum granulosum, where pH is neutral. The neutral pH changes to more acidic pH of 4.5-5.5 in the stratum corneum where the mentioned proteases are activated to perform their proteolytic function in a cascade manner. The KLK 5 is able to autoactivate itself and activate other KLKs such as KLK-7 and 14. Although KLK-5 is capable of enzymatic activity at neutral pH of the stratum granulosum, the LEKTI in their vicinity inhibit their function (123).

In a recent study, Zhu et al. described that persistently upregulated KLK-5, independent of PAR2, stimulate secretions of IL-8, IL-10, and TSLP-1 (124). In another study, the mass level of different KLKs were measured in healthy and AD patients. Although mass volume of KLK-7 and KLK-11 were increased in AD skin, the KLK-5 mass volume remained unchanged (125). Lastly, reduced function of LEKTI, due to 420K variant, has been observed in the AD, further explaining the decreased inhibitory action against KLK-5 (126).

Variations in genes encoding for proteases such as KLK-7 and their inhibitors such as serine peptidase inhibitor Kazal type 5(SPINK- 5), increase the proteolytic activity in the stratum corneum and subsequently cause barrier disruption and dysfunction (127, 128). Furthermore, FLG gene variants with their resulting reduction of NMF production may increase the pH, which facilitates the activation of protease cascade (129). Finally, exogenous proteases, such as staphylococcal proteases, or other barrier aggravating agents such as soaps and detergents may alter the balance between exogenous and endogenous proteases and their inhibitors, ending in excessive desquamation and degradation (130).

Stratum corneum is made of a combination of lipids such as cholesterol, ceramides, and free fatty acid. Altered stratum corneum lipids were documented in patients with AD. The ceramides in stratum corneum are critical for skin barrier function and prevention of TEWL. There are number of mechanisms attributed to ceramides reduction in AD. Current research shows that there is a disturbance in the length of the fatty acid chains of ceramides in patient with AD, where the amount of long chained fatty acid ceramides is decreased and the short chained fatty acid ceramides are increased. The length of fatty acid chain of ceramides is inversely proportional to the TEWL and barrier function of the skin. In addition, there is a significant correlation between disease severity and changes in lipid composition and organization in AD (131). It is documented that loricrin and involucrin, which are protein component of skin barrier, are significantly decreased in both acute and non-lesioned skin of AD subjects, further proving the flaw in the skin barrier of AD patients (132).

Tight junctions (TJs) are groups of adhesive proteins which are placed on opposing membranes of keratinocytes in stratum granulosum. They are crucial in maintaining skin barrier integrity as they control the passage of fluids and solutes paracellularly acting as a selective barrier. They may also be recognized as a second physical barrier beneath the stratum corneum. Claudin-1 and claudin-23 are among the most affected TJs proteins in AD. In a study conducted by De Benedetto et al. on expression or function of TJ proteins such as claudin-1 in epithelium of AD and non-atopic subjects, a striking reduction in expression of TJ proteins in AD subjects were observed. The expression of claudin-1 inversely correlated with Th 2 lymphocyte polarization biomarkers such as serum total IgE and total eosinophil count (133). In another study on animal models of AD, it was revealed that upregulation of claudin-1 attenuated the severity and natural course of AD (134). Further, it was observed that cytokines produced by Th-2 and Th-17 lymphocytes, such as IL-17, reduce the expression of tight junction proteins claudin-4 and 8 in AD patients (135). Reduced expression of tight junction proteins such as claudin-1 and 4 have been observed during infection with pathogenic strains of Staphylococcus. During infection, the TJs are primarily promoted to maintain barrier function although later their expression is reduced (136).

## Atopic dermatitis treatments; conventional to novel delivery systems

The chronic course of AD is characterized by periods fluctuating between remission and exacerbation. The exacerbation periods are also known as flares. The flares are crucial in dosing of any treatment, as in these periods’ patients may require intensified doses. In many cases patients and caregivers judge the success of the treatment according to the frequency and severity of the flare-ups, hence it is crucial to educate patients regarding signs and symptoms of flares to improve the adherence and compliance with treatment and subsequently the treatment outcome. There are two approaches in long term management of AD with chronic tendency. The approaches are denoted as reactive or proactive. The reactive approach involves daily moisturizing and skin hydration plus an anti-inflammatory of choice. Conversely, proactive management involves intermittent application of anti-inflammatories and emollients to affected, newly affected or unaffected areas of skin. The effectivity of proactive management in controlling flare-ups has been shown in multiple studies, hence it is recommended to patients who are experiencing symptoms of moderate to severe AD with compromised quality of life (137).

The first understanding and treatment of eczema dates to 400 BC, when Hippocrates described AD as a cutaneous manifestation of internal diseases or a disequilibrium in humor. In that era, it was believed that symptoms of eczema, such as oozing or itching, should not be treated promptly as it may mask or worsen the internal disease (138). Nearly a thousand years ago Avicenna, a Persian polymath, accumulated medical knowledge known to human about eczema into a comprehensive text called Canon. The proportional relation between long bathing and dry skin was described in an anecdote, recommending shorter baths to prevent eczema (139).

The ancient therapeutic approaches focused on the cure of the internal disease as recommended by Hippocrates. Patients applied mercury ointments near lesions to form a blister, or ingested sulfur or arsenic to treat the internal disease. Leeches, body wrapping in rubber or laxatives were also used as detoxifying methods to clear eczema. Later an Austrian dermatologist, Hebra, described eczema as a solo entity with chronic course which could not be cured fast and recommended topical application of soaps and oils and other natural lotions to treat it (140).

In 1933, AD was the new term introduced for description of eczema. Along with the change in the terminology, new therapies emerged in late 19^th^ century. Physicians understood the role of food in manifestation of AD, and recommended food avoidance in patients with positive skin tests. Further, they understood that first symptoms could manifest very early even in infants who are exclusively breastfed. In 1952, the use of compounds containing hydrocortisone was proposed. The introduction of corticosteroids revolutionized management of AD (141). Nowadays, with a rise in so-called steroid phobias, many are going back to primitive remedies. The modern practice has added multiple effective therapies to topical corticosteroids.

Non-medical treatments for AD are essential in managing this chronic skin condition by addressing triggers and contributing factors that can exacerbate symptoms. These non-medical approaches primarily focus on skincare practices and lifestyle modifications. Gentle skincare routines, which include the use of fragrance-free, hypoallergenic moisturizers, and mild non-soap cleansers, are essential to maintain skin hydration and prevent flare-ups. Avoiding harsh soaps, hot water, and excessive scrubbing can help protect the skin’s natural barrier. Lifestyle modifications often involve identifying and minimizing environmental triggers such as allergens, irritants, and stress. Implementing dietary changes and stress-reduction techniques like meditation or yoga may also complement medical treatments, offering individuals with AD a holistic approach to managing their condition and improving their quality of life (142).

Corticosteroids are commonly used as the first-line treatment of AD, offering relief from itching, inflammation, and redness through inhibiting antigen processing by dendritic cells and Langerhans cells and production of inflammatory cytokines (143). Among the most frequently prescribed corticosteroids for AD are hydrocortisone, triamcinolone, and betamethasone. These medications are available in various forms, including creams, ointments, and lotions, providing flexibility in application. Treatment regimens should be individualized with lower-potency corticosteroids, such as hydrocortisone 1%, preferred for the face and neck and more potent options, such as clobetasol, for other areas of body. A thin layer is applied to the affected skin once or twice daily followed by a moisturizer, gradually tapering as symptoms improve to minimize potential side effects like striae, atrophy or skin thinning (144). Hydrocortisone-loaded poly ϵ-caprolactone nanoparticles (PCLNPs) in ointment form, have showed better permeation and controlled release with no change in the toxicity compared to the conventional dosage form (145). Betamethasone-17-valerate prepared under hot high-pressure homogenization in the form of solid lipid nanoparticles (SLNs) have also shown greater depot in both intact and impaired skin barrier compared to the conventional ointments (146). Additionally, clobetasol propionate has been nanotechnologically formulated *in vivo*, to a nano-emulsion composed of eucalyptus oil and surfactant with improved solubility which reduces edema notably (147).

Topical calcineurin inhibitors (TCIs), such as tacrolimus and pimecrolimus, are important non-steroidal options as the second line of treatment for AD. They function by reducing calcineurin-dependent T lymphocytes activation and inhibiting genes coding for transcription of inflammatory cytokines. These medications are especially useful in sensitive areas such as face and skin folds, where corticosteroids may pose risks of atrophy or thinning of the skin. Tacrolimus is available in ointment form, while pimecrolimus comes in form of cream. The typical regimen involves applying a thin layer of the medication to affected areas twice daily with special care regarding the age of the patient, as tacrolimus 0.1% should only be prescribed to patients older than 15 years. Among usual side effects of the use of TCIs are skin burning and itching (148). Tacrolimus ointments prepared nanotechnologically with ionic gelation method has shown improved permeation at reduced doses. The hydrophilic nature of chitosan and nicotinamide increase the entrapment efficiency of hydrophobic tacrolimus, resulting in greater permeation and retention (149). Further, polymeric nanoparticles of tacrolimus decorated by hyaluronic acid formulated under high pressure homogenization-evaporation method has shown targeted sustained release pattern compared to previous formulations (150). Other nanotechnologically designed tacrolimus, such as thermosensitive SLNs or microemulsions have also shown better penetration and retention in the skin. The thermosensitive SLNs showed penetration to up to 500 μm compared to ointment penetration of 150 μm *in vivo*, which suggest high drug loading efficiency (151).

Although the itch associated with AD is mostly of non-histaminic origins, antihistamines of the first and the second generations may also be prescribed to patients who experience problems with sleeping or scratching during sleep. As first-generation antihistamines, such as diphenhydramine or hydroxyzine, have sedative characteristics and may not be a reasonable option for patients at work or school, the use of oral second-generation antihistamines, such as cetirizine or levocetirizine, is advised. Recently, levocetirizine and cetirizine hydrochloride have been nanotechnologically formulated in gel form, available for topical use to prevent itch and erythema. The niosome and chitosan nanoparticles ensure optimized skin retention (152).

Systemic immunosuppressive agents such as cyclosporine, azathioprine, and methotrexate are added to the management when conventional treatments prove insufficient. Barbosa et al., developed a fucoidan/chitosan nanoparticle for topical treatment by methotrexate. The formulation ensures a strong inhibition of pro-inflammatory cytokines production along with safe drug delivery to deeper layers of skin (153). Additionally, Verma and Fahr utilized a lipid mixture to develop a vesicle for enhanced topical delivery of cyclosporin A. The study proved effective delivery dependent on concentration of ethanol and size of the vesicles (154). These medications work by dampening the overactive immune response responsible for the skin inflammation characteristic of AD. These drugs are usually prescribed in case of treatment- resistant AD or very severe AD. Patients who are candidate to receive systemic therapies should undergo pre-treatment renal and hepatic screen, as these medications predispose to kidney or liver dysfunction (155).

Many patients with AD do not achieve adequate symptom control with conventional treatments, leading to persistent discomfort and reduced quality of life. Patients may have varying responses to conventional treatments, emphasizing the need for more tailored and effective therapies. A better understanding of the immunological and genetic background of AD has highlighted the potential targets for novel therapies that can address the disease’s root causes more effectively. Dupilumab is an FDA-approved breakthrough treatment for AD. This monoclonal antibody targets IL-4 and IL-13 signaling pathway, inhibiting Janus kinase/signal transducers and activators of transcription (JAK-STAT) pathway of signaling. Patients typically receive dupilumab as a subcutaneous injection every two weeks, and it is often used in conjunction with other therapies (156). One of the side-effects associated with the use of dupilumab is conjunctivitis. The eye redness accompanied by burning and foreign body sensation was reported by 28% of patients (157). Janus kinase (JAK) inhibitors and phosphodiesterase-4 (PDE-4) inhibitors represent promising novel therapeutic approaches in the treatment of AD, addressing the underlying inflammatory processes. PDE4 is a key player in generations of cytokines such as IL-4 and IL-13, subsequently its inhibition in animal models have shown immunosuppression by increasing AMP and reducing IL-4 and TNFα (158). JAK inhibitors target the Janus kinases which are key signaling pathways in cytokine productions bridging various cytokine receptors with STAT transcription factors. IL-2, IL-4 and many other cytokines are expressed on basis of such signaling, thus JAK inhibitors could be a promising therapy for AD. Tofacitinib is an example of a JAK-1 and 3 inhibitors found to be effective in oral and topical form against AD (159).

In a recent clinical trial, the safety and efficacy of etrasimod, an oral selective sphingosine-1-phophate receptor _1,4,5_ modulator, was assessed. Etrasimod regulates the migration of lymphocytes and modulates migration of dendritic cells to lymph nodes. Further, etrasimod prevent the release of certain subsets of lymphocytes from lymphatic tissues, reducing the final number of available lymphocytes for recruitment to the site of inflammation. Although percent changes in EASI, and improvements in validated Investigator’s Global Assessment score were observed in patients receiving etrasimod 2-mg compared to the placebo group, the primary outcome of EASI-75 was not met (160).

Further, Rocatinlimab, an anti-OX40 antibody for subcutaneous injections, was evaluated in a phase 2b, randomized, placebo-controlled clinical trial. The trial showed a significant reduction in the EASI of patients receiving the treatment compared to the placebo group. The clinical response was maintained twenty weeks after the treatment cessation. The OX40 is crucial in the expansion and survival of T lymphocytes and subsequent memory formation, making anti-OX40 a great candidate for the treatment of moderate to severe AD (161).

While extensive research has been conducted in the field of dermatology and the immunological aspects of AD, numerous questions are left without answers regarding pathogenesis and treatment of this condition. The advances in the field of nanotechnology and the introduction of novel topical and biological therapies have brought about significant advancements, substantially enhancing patient care, and elevated the quality of life for those affected. Although progress has been made, there remains an untouched area of more individualized treatments and identification of specific types of biomarkers, such as serum or plasma biomarkers. This unexplored area promises a fresh avenue for monitoring the disease’s progression, comprehending its trajectory, and assessing the effectiveness of appointed therapies of different characteristic. The potential discovery of such biomarkers could revolutionize the way we understand pathogenesis of AD, leading to better outcomes for patients and improved therapeutic approaches as well as early diagnosis and prevention.

## Author contributions

MA: Writing – original draft, Writing – review & editing, Conceptualization, Investigation. MK: Writing – review & editing. MR: Writing – review & editing. JC: Supervision, Writing – review & editing. JK: Funding acquisition, Supervision, Writing – review & editing.

## Glossary

**Table:**

AD	atopic dermatitis
DALYs	disability-adjusted life-years
EASI	eczema area and severity index
SCORAD	SCORing Atopic Dermatitis
AMP	antimicrobial peptide
TLR	toll-like receptor
INF-γ	interferon-gamma
IL	interleukin
LC	Langerhans cells
PRR	pathogen recognition receptor
PAMP	pathogen associated molecular pattern
HLA	human leukocyte antigen
MHC	major histocompatibility complex
DAMP	damage associated molecular pattern
TNF-α	tumor necrosis factor-alpha
dDCs	dermal dendritic cells
NK	natural killers
TGFβ	transforming growth factor β
TSLP	thymic stromal lymphopoietin
GIT	gastrointestinal tract
TEWL	trans-epidermal water loss
NMF	natural moisturizing factor
FLG	filaggrin
NF-κB	nuclear factor kappa B
RSV	respiratory syncytial virus
HSV	herpes simplex virus
SpA	Staphylococcal protein A
KLK	kallikrein-related peptidase
PAR2	protease activated receptor 2
LEKTI	lympho-epithelial Kazal type related inhibitor
SPINK5	serine peptidase inhibitor Kazal type 5
TJ	tight junction
PCL	poly ϵ-caprolactone
NPs	nanoparticles
SLNs	solid lipid nanoparticles
TCI	topical calcineurin inhibitor
JAK-STAT	Janus kinase/signal transducers and activators of transcription
PDE	phosphodiesterase
